# Parenthood and Cross-Border Surrogacy: What Is ‘New’? The ECtHR’s First Advisory Opinion

**DOI:** 10.1093/medlaw/fwz042

**Published:** 2020-02-13

**Authors:** Alice Margaria

**Affiliations:** Department of ‘Law and Anthropology’, Max Planck Institute for Social Anthropology, Advokatenweg 36, 06114 Halle/Saale, Germany

## I. INTRODUCTION

On 4 October 2019, the French Cour de Cassation finally put an end to the struggle of Sylvie and Dominique Mennesson, and their twins who were born from a gestational surrogacy arrangement in California in 2000. It delivered a verdict that the family had expected for 19 years: it validated the transcription into the French civil registry of the foreign birth certificates that designate Mr and Mrs Mennesson as the father and the mother of their daughters Fiorella and Valentina.^1^ This article focuses on and examines a particularly decisive stage in the Mennessons’ crusade to justice: the Advisory Opinion delivered by the European Court of Human Rights (‘the Court’ or ‘the ECtHR’) on the issue of recognition in domestic law of the legal relationship between a child born through gestational surrogacy abroad and the intended mother.^2^ Against the background of the Court’s previous case law on surrogacy, this Opinion is herein approached as a further opportunity to (re)consider what kind of link—ie genetic, gestational, and social/intentional—makes someone a legal mother in the era of reproductive technology. Being the first of its kind, this Opinion offers the chance to reflect also on the advisory mechanism itself, and what it can concretely add to the Court’s jurisprudence.

This article is divided into five sections. Section II introduces the Court’s involvement in the area of cross-border surrogacy and the ‘new’ advisory mechanism available under Protocol No 16.^3^ In Section III, the focus shifts on the first Advisory Opinion issued by the Grand Chamber, in particular on the context against which it originated. Section IV is devoted to outlining the Opinion’s content, starting from the preliminary considerations made by the Court and followed by the latter’s response to the two questions raised by the French *Cour de Cassation*. Sections V and VI include the analytical core of this article and engage with two distinct yet interrelated aspects. Section V reflects on the way the Opinion has been drafted and argues that it realises a ‘good compromise’^4^ between the—often perceived as competing—approaches of individual justice and constitutional justice. Despite its focus on the *Mennesson* case, the Court offers indeed also some guidance on how to deal with future cases. The content of such guidance is dissected in Section VI, which brings to the fore the ‘construction’ of motherhood emerging from the Advisory Opinion. It will be concluded that, despite remaining anchored to the idea that biology is to be privileged, this Opinion reveals the Court’s awareness that being a mother—and more generally, a parent—entails much more and sometimes something different from contributing with one’s own genetic material to conception and/or gestating a pregnancy.

## II. THE ECtHR AND CROSS-BORDER SURROGACY: INTRODUCING THE ‘NEW’ ADVISORY MECHANISM

Judges have been compared to ‘certified architects or engineers of the New Biology’.^5^ Bioethics is indeed an area in which the judiciary has long since played—and continues to play—a driving role in moulding the legal response to new social realities created as a result of scientific progress.^6^ Owing to exigencies of time, written laws might become largely obsolete or even lead to absurd outcomes when applied to circumstances that were unforeseeable at the time of drafting. New laws directed to regulate new situations in contemporary society tend to be enacted with a delay and—whenever in place—are often the result of compromises between diverging cultural, political, and other social forces, thus eventually leaving room for judicial interpretation. It is therefore not surprising that, *vis-à-vis* disputes concerning bioethical issues, judges are often called to ‘fill the void of indecisiveness’.^7^

This dynamic can be observed also within the domain of surrogacy and, more specifically, with respect to the controversial implications of cross-border arrangements on the establishment of legal ties between the child and the intended parents. Apart from troubling national judges in Europe and beyond, the issue of whether and how parent–child relationships created through surrogacy arrangements abroad ought to be legally recognised has also reached the ECtHR. In light of the significant impact that its decisions have had on the legislation of the Contracting States, the Court is viewed as one of the most influential institutional actors not only in the creation of European family law^8^ but also in the production of what has become known as ‘biolaw’.^9^

It follows from the ‘living instrument’ approach as one of its main interpretative doctrines that the Court has been called to reflect on the repercussions of social and technological progress on human rights in various fields—including that of reproductive rights—on several occasions. Considering the sensitive moral and ethical issues that arise ‘against a background of fast-moving medical and scientific developments’,^10^ the Court has generally acknowledged a particular need for restraint and proved overall hesitant to interfere with national policies concerning bioethical issues.^11^ In relation to surrogacy-related claims, however, the stance adopted by the Court has been less cautious than expected. On the specific issue of the recognition of intended parenthood following surrogacy, the Court has exercised its role of ‘shaping rights’^12^ through a double channel. Not only has this issue been dealt within the Court’s contentious case law, but it has also been at the core of its first Advisory Opinion adopted under the new Protocol No 16.

This Protocol opens up the possibility for the ‘highest domestic courts’ to request the Court to deliver an Advisory Opinion on ‘questions of principle relating to the interpretation or application of the rights and freedoms defined in the Convention or the protocols thereto’.^13^ In the wake of the concern shown by Government leaders of the Contracting States at the Brighton Conference,^14^ this advisory function has been designed to improve the functioning and long-term effectiveness of the system of the European Convention on Human Rights (ECHR) by pursuing two fundamental objectives: enhancing the interaction between the Court and national courts^15^ and alleviating the Court’s caseload. The underlying *rationale* is that the advisory function could serve as a ‘pre-emptive standard setting mechanism’^16^: if the Court succeeds in providing guidance to national courts in interpreting and applying Convention standards, it is expected that a higher number of cases could be easily solved at the national level.

Apart from being the last Contracting Party to ratify Protocol No 16 and thus to determine its entry into force, France is also the first jurisdiction to have made use of this procedure. In the face of a (further) legal initiative by Mr and Mrs Mennesson—applicants of the well-known case against France decided by the ECtHR in 2014^17^—the French *Cour de Cassation* has consulted the Grand Chamber to obtain its views on the recognition in domestic law of a legal parent–child relationship between a child born through a gestational surrogacy arrangement abroad and the intended mother. The following section will retrace the development of the *Mennesson* case from its outset.

## III. BACK TO THE ORIGINS: HOW DID IT ALL START?

France is among the European States where surrogacy is explicitly prohibited under the law. In practice, national bans or restrictions on the use of surrogacy and other assisted reproductive technologies (ARTs) have proved rather ineffective in preventing intended parents from accessing the sought services and attaining their reproductive goals abroad. The widespread discontent and prohibition of surrogacy—coupled with the entrenchment of the principle *mater semper certa est*—has however resulted in the so-called ‘new illegitimacy’ in Europe.^18^ Although turning domestic prohibitions into a ‘dead letter’,^19^ resorting to foreign reproductive markets has indeed often exposed intended parents to difficulties in having their parental status lawfully established abroad recognised at home and, as a result, led to disparate treatment of children born out of international surrogacy arrangements.

This Advisory Opinion originates from the story of one of these couples and their twin daughters born through gestational surrogacy in California using the intended father’s sperm and donor eggs. The twins had been identified in the USA as the children of Mr and Mrs Mennesson. Once they arrived in France, they were allowed to live together but nonetheless denied recognition of their parent–child relationships as it would be contrary to public policy. After exhausting national remedies, the Mennesson family lodged an application with the ECtHR. They submitted that the children’s best interests had been disregarded and complained in particular of a breach of their right to respect for private and family life guaranteed by Article 8 ECHR. In its 2014 judgment, whilst ruling out any interference with the right to respect for family life,^20^ the Court found that the refusal to recognise intended parenthood affected the children’s ability to establish the essence of their identity—that includes parentage—thus violating their right to respect for private life.^21^ Because of the biological connection existing between the children and their intended father, these considerations assumed—in the Court’s view—a ‘special dimension’.^22^

This ruling has been described as ‘a watershed moment for the regulation of international surrogacy in Europe’^23^ in light of its immediate effect on domestic law. In France, the *Cour de Cassation* tempered its position allowing for the registration of a foreign birth certificate into the French civil register, unless evidence suggests that the document is irregular, falsified and that the facts recorded do not reflect biological reality.^24^ The recognition of intended parenthood became therefore possible in so far as the birth certificate designated the intended father as the child’s father where he had a genetic connection with the child. Intended motherhood was, however, left out in the cold. The *Cour de Cassation* reiterated its commitment to the principle *mater semper certa est* and, therefore, parturition as the only criterion to determine legal motherhood.^25^ Only 2 years later, it clarified that the intended mother could nonetheless seek to adopt the child, provided that the statutory requirements were met and adoption was in the child’s best interests.^26^

The case of *Mennesson* came again under the spotlight in 2018, when the applicants took advantage of the recently established procedure that allows for the review of decisions issued by the Cour de Cassation that have been declared in violation of the ECHR by the Strasbourg judges. On 16 February 2018, the French Civil Judgments Review Court accepted their request for reviewing the previous refusal to register the foreign birth certificates. Whilst it considered the issue of the recognition of the biological father’s parental relationship with his twin daughters settled, the *Cour de Cassation* expressed doubts as to the degree of the margin of appreciation left to the States with respect to the parental status of the intended mother. Hence, the request to clarify the effects and consequences of the ECtHR’s previous jurisprudence on the position of the intended mother through the following two questions:

1. By refusing to enter in the register of births, marriages and deaths the details of the birth certificate of a child born abroad as the result of a gestational surrogacy arrangement, in so far as the certificate designates the ‘intended mother’ as the ‘legal mother’, while accepting registration in so far as the certificate designates the ‘intended father’, who is the child’s biological father, is a State Party overstepping its margin of appreciation under Article 8 of the European Convention for the Protection of Human Rights and Fundamental Freedoms? In this connection should a distinction be drawn according to whether or not the child was conceived using the eggs of the ‘intended mother’?

2. In the event of an answer in the affirmative to either of the two questions above, would the possibility for the intended mother to adopt the child of her spouse, the biological father, this being a means of establishing the legal mother-child relationship, ensure compliance with the requirements of Article 8 of the Convention?^27^

## IV. THE CONTENT OF THE OPINION

### A. Preliminary Considerations: Setting the Boundaries

Before addressing the two questions posed by the *Cour de Cassation*, the Grand Chamber makes some preliminary considerations in order to delimit the boundaries of its advisory function and, more specifically, of its Advisory Opinion. The Court identifies three sets of limits. First, it explains that the scope of the procedure is ‘not to transfer the dispute to the Court’,^28^ but rather to provide guidance on issues related to the Convention to the requesting court or tribunal when settling the case pending before them.^29^ It follows that the Court does not engage with the facts of the case, as it would do under its contentious jurisdiction, and does not rule on the outcome of domestic proceedings.^30^ Rather, as clarified by the Court, it falls on the requesting national court or tribunal to draw conclusions from the Grand Chamber’s Opinion and to solve the matter pending before them.^31^

Secondly, according to the Court, the Opinion must be limited to ‘points that are directly connected to the proceedings pending at the domestic level’.^32^ The Grand Chamber anticipates therefore that its Opinion will not deal with situations ensuing from procreative paths different from that undertaken by Mr and Mrs Mennesson. More specifically, it formally excludes situations involving traditional surrogacy arrangements—that is when the child was conceived using the eggs of the surrogate mother^33^—or arrangements involving the genetic material of the intended mother,^34^ from the scope of its Opinion. Thirdly, and finally, the Court takes the time to specify that its Opinion will not address either the right to respect for family life of the children and intended parents or the latter’s right to respect for private life.^35^ At least *prima facie*, therefore, the approach taken by the Grand Chamber seems to be that of restricting its views to the elements that appear strictly necessary to decide the case pending before the *Cour de Cassation*.

### B. Issue 1: Recognition?

The first issue raised by the *Cour de Cassation* concerned whether Article 8 required national authorities to recognise the legal relationship between the intended mother and the child born from surrogacy lawfully established abroad. To address this question, the Grand Chamber identifies two factors as particularly weighty: the child’s best interests and the scope of the margin of appreciation enjoyed by State Parties.^36^ With respect to the former, the Advisory Opinion reiterates that the detrimental impact of non-recognition is not limited to the parents, but affects also the children, who are consequently placed in a position of legal uncertainty regarding their identity within society.^37^ The Court explains that the lack of recognition of the relationship between a child born through a surrogacy agreement abroad and the intended mother might be disadvantageous in many respects: children might be denied their intended mother’s nationality; they might experience difficulties in remaining in their intended mother’s country of residence as well as in preserving their relationship with their mother in case of parental separation; their inheritance rights might be curtailed; and, finally, they might be left with no protection in case the mother does not take care of them.^38^

Whilst acknowledging the ‘risks of abuse’^39^ that surrogacy arrangements trigger as well as the importance of the right to know one’s origins, the Court is of the view that:

the general and absolute impossibility of obtaining recognition of the relationship between a child born through a surrogacy arrangement entered into abroad and the intended mother is incompatible with the child’s best interests, which require at a minimum that each situation be examined in the light of the particular circumstances of the case.^40^

Moving onto discussing the margin of appreciation enjoyed by the State, this Opinion has been effectively described as ‘a child of its time’.^41^ In the so-called ‘age of subsidiarity’^42^—and, moreover, given the framing of the question by the *Cour de Cassation*—it is not surprising that the margin of appreciation is given a central role in the Advisory Opinion. On the wake of its previous judgments, the Court recalls that—on the basis of its comparative-law survey—there is no European consensus on whether intended parenthood following surrogacy ought to be recognised.^43^ This situation would, in line with the Court’s practice, lead to a wide margin of appreciation.^44^ The Court concludes, however, that the margin ought to be reduced because the issue at stake involves particularly important facets of an individual’s identity as well as ‘essential aspects of the (children’s) private life’.^45^

Thus, given the requirement of the child’s best interests and the narrow margin of appreciation afforded to Contracting States in such situations, the Court is of the opinion that Article 8 ought to be read as requiring domestic law to allow for the recognition of the parent–child relationship lawfully established abroad between the child and the intended mother, who is designated as the ‘legal mother’ on the birth certificate.^46^ Prior to addressing the second question posed by the *Cour de Cassation*, the Court finds it important to add that, in the hypothetical scenario where the intended mother is genetically linked to the child, the need to provide for such recognition ‘applies with even greater force’.^47^ In so doing, it ends up partially ignoring its own disclaimer (Section IVA) by providing some guidance—even if through an *obiter dictum*—on a situation that is different from the one pending before the national court.

### C. Issue 2: What Kind of Recognition?

Having clarified that the right to respect for private life of the children born abroad from gestational surrogacy entails the possibility for the intended mother–child tie to be legally recognised, the Court goes on to examine the *quo modo* of such recognition. Whilst stressing that the uncertainty surrounding the children’s status should be ‘as short-lived as possible’,^48^ the Court argues that the choice of means by which to enable recognition falls within the State’s margin of appreciation.^49^ This conclusion is grounded on the lack of a European consensus also on this issue, and on the fact that the children’s identity is perceived as ‘less directly at stake’^50^ when the issue is *how* to implement the duty of recognition. In the Court’s view, therefore, Article 8 does not require the recognition of the mother–child relationship *ab initio*, but ‘at the latest when it has become a practical reality’^51^ based on the assessment of national authorities. As a result, the Court does not consider the entry of the foreign birth certificate into the register of births, marriages, and deaths as the only acceptable form of recognition.^52^ Adoption by the intended mother represents—in the Court’s opinion—a valid alternative, provided that national legislation enables a decision to be taken ‘promptly and effectively, in accordance with the child’s best interests’.^53^

As summarised by Lavrysen, therefore, the sole obligation that the Opinion prescribes is to provide ‘access to an effective procedural mechanism’^54^ that allows for the recognition of the legal relationship between the child and the intended mother, if this is in line with the assessment of the child’s best interests in light of the circumstances of the case. The guidance offered by the Grand Chamber—whose ‘limited’^55^ scope and potential effects in practice had been questioned^56^—has however proved sufficient to put an end to the legal battle of the Mennesson family. On 4 October 2019, the *Cour de Cassation* decided in favour of the recognition of the legal relationship between Mrs Mennesson and her twin daughters.^57^ Most importantly, it ruled out adoption as an unsatisfactory option in this specific ‘case that had been ongoing for more than 15 years’,^58^ and held that the recognition of the foreign birth certificates that designate Mrs Mennesson as the ‘legal mother’ of the twins can no longer be denied.

## V. DRAFTING TECHNIQUES: A ‘GOOD COMPROMISE’

Having outlined the content of the Opinion, this section scrutinises the drafting techniques used by the Court with the view of examining the actual reach of its intervention. International human rights law does not offer a ‘univocal answer’^59^ and ‘may be interpreted to allow both permissive and prohibitionist national approaches to the regulation of international surrogacy’.^60^ The complexity of the human rights discourse in the domain of surrogacy is visible also in the ECtHR jurisprudence, where the impact of cross-border arrangements on legal parenthood has given rise to diverging views within the Court. Particularly emblematic is the reversal of the Chamber’s decision by the Grand Chamber in the case of *Paradiso and Campanelli v Italy*. In this case, the child—born out of a surrogacy arrangement in Russia—had been placed for adoption in light of a DNA test showing no biological connection with the intended parents. Whilst the Chamber considered the child’s removal from Mr Paradiso and Mrs Campanelli in breach of their right to respect for family life,^61^ the Grand Chamber found it justified by the need to prevent an illegal situation put in place by the intended parents from being legalised and, therefore, compatible with Article 8.^62^ Not only have the Chamber and the Grand Chamber reached opposite outcomes relying on substantially different reasoning; they have both issued judgments that, far from being endorsed by all judges sitting on the Court, have given rise to concurring and/or dissenting opinions.

Disagreement is a possibility also under Article 4(2) of Protocol No 16: ‘If the advisory opinion does not represent, in whole or in part, the unanimous opinion of the judges, any judge shall be entitled to deliver a separate opinion.’ Despite so, the Grand Chamber did not—in its advisory capacities—take advantage of this opportunity. Possibly motivated by the desire to strengthen its authority, the Court delivered a first, unanimous Advisory Opinion. At the same time, however, if regard is given to the way the Opinion is drafted, the Court seems to hide the inevitable difficulties to express views on a particularly controversial issue—like that at stake—behind procedural caution. Despite acknowledging that the value of Advisory Opinions consists also ‘in providing national courts with guidance on questions of principle relating to the Convention applicable in similar cases’,^63^ the Court then chooses—at least formally—to tailor its reasoning around the specific case. In addition to the boundaries set in its preliminary considerations (Section IVA), the Court does not miss a chance to remind us of the peculiarities of the *Mennesson* case and to state that it will ‘limit its answer accordingly’,^64^ also when addressing the two issues raised by the *Cour de Cassation*. The Court’s multiple attempts to restrict the scope of its Opinion can, therefore, be read as an externalisation of its awareness of the delicate complexity of the issue at stake: not just in the merits (ie ethical and moral dimensions of surrogacy), but also as far as the Court’s relationship with national authorities is concerned.

On the one hand, the prudent attitude shining through the Opinion is in line with the requirement set by Article 1(2) of Protocol No 16, according to which an Advisory Opinion can be requested ‘only in the context of a case pending’ before the requesting national court or tribunal. On the other hand, however, it has been rightly pointed out that there is no provision in the Protocol that states that the Court has to similarly confine its response to such context.^65^ The Court’s choice to restrict its views to the context of the *Mennesson* case has been considered even ‘at odds with’^66^ Protocol No 16’s general aim of to provide guidance on questions of principle related to the interpretation and application of the Convention.^67^ The idea underlying this critique is that, if the Advisory mechanism is truly expected to reduce the Court’s caseload, the ECHR system would rather benefit from Opinions that transcend the specific case and anticipate foreseeable difficulties that might emerge in similar future cases.

While sharing the above concerns, it is herein argued that the Court’s first Advisory Opinion ought to be appreciated as it contributes, in its current form, to realising the Court’s ‘twin role’.^68^ In other words, it provides—thus showing that it is possible—a ‘good compromise’^69^ between the Court’s long predominant duty to deliver individual justice and the ‘constitutional role’ Protocol No 16 aims to strengthen. These two functions have been often perceived as being in tension. On the one hand, the individual justice approach is premised on the view that the right of individual petition represents the core of the Convention system, the ‘Crown jewel of the Convention’.^70^ The Court’s ‘primary’ duty consists therefore in deciding each application on a case-by-case basis with the purpose of ‘provid(ing) individual relief’ to those affected.^71^ On the other hand, the notion of ‘constitutional justice’ in the ECHR context has been first used by the Court’s then President, Luzius Wildhaber. He envisaged a ‘constitutional future’^72^ for the ECtHR, where the latter would adjudicate ‘essentially public-policy issues’,^73^ focus on ‘decisions of “principle”, decisions which create jurisprudence’,^74^ thus developing the standards and clarifying the substantive content of the ECHR. The Court itself, when discussing how Protocol No 16 would enhance the Court’s ‘constitutional’ role, explains that: ‘Advisory opinions provide an opportunity to develop the underlying principles of law in a manner that will speak to the legal systems of all the Contracting Parties. … The procedure would thus allow the Court to adopt a larger number of rulings on questions of principle and to set clearer standards for human rights protection in Europe.’^75^

The most tangible evidence of the ‘good compromise’ set by the Court’s first Advisory Opinion between these two approaches to justice is paragraph 47. Despite observing (once again) that national proceedings do not concern a situation where the child born from surrogacy has been conceived with the eggs of the intended mother, the Court is unable to resist the temptation to comment on that scenario too: ‘the need to provide a possibility of recognition of the legal relationship between the child and the intended mother applies with even greater force in such a case’. Possibly helped by the way the *Cour de Cassation* framed its first question, the Court seizes the occasion to go ‘hors du cadre’,^76^ and to express its views on a situation that is different from the *Mennesson* case, yet likely to emerge in future cases. In so doing, the Court shows itself willing not only to engage in a ‘cercle vertueux du dialogue’^77^ with national courts but also to take up a role of ‘normative guidance’^78^ that goes beyond the single case. More specifically, it seems that the Court uses its advisory jurisdiction also to induce general principles from the specific case pending before the national court. In this particular instance, the Court’s role of guidance translates into expressing its normative visions on the relevant factors to determine legal motherhood following surrogacy. In so doing, the Grand Chamber centres its Opinion on the *Mennesson* case, whilst still giving Contracting States ‘some pointers’^79^ for regulation. What can be learned in view of future cases is examined in the following section.

## VI. MOTHERHOOD AND SURROGACY

Regardless of their diverse approaches to surrogacy, legislative frameworks across Europe follow the rule *mater semper certa est* to define who is a mother. In other words, legal motherhood is determined according to the traditional biological/gestational criteria: the woman who is pregnant and gives birth to the child is treated as the child’s mother. The attribution of the paternal status is governed by a different presumption—the so-called ‘marital presumption’—that identifies the husband of a woman giving birth to a child as the father of the child. In practice, however, surrogacy entails something different. The woman who gestates the child is not the same as the woman who raises the child. Moreover, the man who acts as a father towards the child is not the husband of the surrogate mother. As such, surrogacy provides legal actors—and society as whole—with precious opportunities of reflection as it questions traditional understandings of who is perceived to be a ‘parent’, especially a ‘mother’.

I have argued elsewhere that, if regard is given to the impact of the wider ARTs-related jurisprudence on fatherhood, the Court has only partially seized the occasion to re-think what makes someone a legal father in present-day family realities.^80^ The Court has indeed started to value the father’s intentions and actual involvement in the child’s life, but only if they are accompanied by other, ‘conventional’ features—in primis, biology.^81^ In *Mennesson*, the biological link between the intended father and his daughters was seemingly the ‘turning point’^82^: the importance of recognising biological parentage formed indeed a significant part of the Court’s reasoning.^83^ A biological understanding of fatherhood runs also through the Grand Chamber’s decision in *Paradiso and Campanelli* that, although finding no violation, ends up reinforcing the previous ruling. The purely social nature of the ties between the intended parents and the child constituted one—or possibly the main—obstacle to qualifying their relationship as ‘family life’.^84^ The ‘radical influence’^85^ of biological unrelatedness went even further to broaden the width of the margin of appreciation enjoyed by the State and, eventually, to entail a rather loose proportionality analysis.^86^

If the Court’s contentious jurisprudence concerning surrogacy contributes to ‘constructing’ a specific image of the ‘father’, the Advisory Opinion brings an interesting contribution to the debate on motherhood. It is certainly remarkable that the Grand Chamber interprets the children’s right to respect for their private life as entailing the possibility of recognition to the advantage of the social ties existing between the intended mother and her daughters born from surrogacy. In so doing, the Court moves beyond the traditional biological/gestational understanding of motherhood and acknowledges the provision of care as another relevant dimension of ‘being a mother’ and, more generally, ‘being a parent’.

Reading Article 8 as demanding a procedure that allows for formal recognition of intended motherhood in domestic law, however, does not amount to place it on an equal footing to biological parenthood. This becomes particularly clear from the Court’s response to the second question raised by the *Cour de Cassation*. The tenacious hold of biology is visible in two respects. First, in the above-mentioned paragraph 47, the Court seems to suggest that the intended mother that has also a genetic connection with the child would be even more entitled than Mrs Mennesson to obtain the recognition of her legal tie with the child born from surrogacy. Secondly, the Court does not impose specific means of recognition for intended motherhood. Differently from biological fatherhood that has to be recognised *ab initio* through the entry into the register of births, marriages, and deaths of the details recorded on the foreign birth certificate, States are left to decide how to fulfil their obligation of recognition *vis-à-vis* intended motherhood. Hence, whilst strengthening the position of the intended mother, this Advisory Opinion does—at the same time—reiterate the privileged role conferred on biology in establishing legal parenthood.

A final aspect of curiosity is that, possibly helped by the facts of the case at stake, motherhood is herein constructed as a derivative of fatherhood. Before addressing the first issue, the Court observes that the question at stake ‘explicitly includes the factual element of a father with a biological link to the child in question’ and ‘it will limit its answer accordingly’.^87^ The Court finds it therefore significant to stress that Mrs Mennesson’s parentage arose in the context of a marriage with the biological father. This aspect of the Advisory Opinion is noteworthy because, traditionally, it has been fatherhood to be understood as a mediated relationship, rather than as an autonomous, direct connection between the father and his child. The ‘marital presumption’ is emblematic of the crucial function that marriage has long played in connecting men to their children. Here, by way of contrast, legal fatherhood is assigned by virtue of biology, and the (marital) connection between the intended parents emerges as one of the factors that make the relationship between the intended mother and her daughters worthy of legal recognition. In other words, the ‘privilege’ of recognition is extended to the intended mother not only as a result of her involvement in the children’s lives but also in her capacity as the wife of the biological father.

An interesting question—potentially for the future—is therefore whether the Court would be willing to adopt the same approach *vis-à-vis* surrogacy arrangements where the intended mother has a genetic link with the child and/or she is the sole intended parent to have such connection. In the latter case, will the provision of care—and the father’s marriage with the mother—be considered sufficient to grant the paternal status to the intended father in a context where biology remains a defining feature of legal fatherhood? Will the biological connection existing between the intended mother and the child constitute a sufficient link in a context where ‘the reproductive experience’ continues to be perceived as a crucial aspect of motherhood?^88^

In conclusion, the Court confirms but, to a certain extent, also questions the primacy of biological parenthood by reading Article 8 as imposing the obligation to enable the recognition of intended motherhood also notwithstanding the rule *mater semper certa est*. This Opinion adds therefore, if not a new block,^89^ some clarity to the Court’s previous jurisprudence on surrogacy: the recognition of the legal relationship between the child and the intended mother is in the child’s best interests because not only biological truth but also relational and emotional aspects contribute to developing one’s private life and personal identity.

## VII. CONCLUDING REMARKS

Advisory Opinions are not binding. Yet, ‘they … form part of the case-law of the Court, alongside its judgments and decisions’.^90^ The interpretative elements offered by the Court under its advisory jurisdiction are therefore analogous in their effects to the interpretation of the Convention adopted by the Court in its judgments and decisions. As such, Advisory Opinions represent an additional vehicle through which the Court exercises its ‘expressive powers’.^91^ In the case at issue, the effects of the Advisory Opinion do not stop at advising the *Cour de Cassation* on how to deal with the national proceedings pending before it, but can be read as going as far as to make statements—and thus to provide guidance—on what elements and values are more important than others in making someone a legal mother. In the Court’s view, it is not just biology and gestation that counts. (Legal) motherhood is—as obvious as it might seem—also about taking care of the child.

The Court’s normative guidance appears particularly welcome in the context of surrogacy and, more generally, when faced with questions at the intersection of family law and bioethics. Despite the progressive emergence of common trends, family law continues to be an introverted subject as it remains particularly open to influence by moral, cultural, religious, social, and political factors^92^—especially so when it has to grapple with advances in medical science. In the area of international surrogacy arrangements, many scholars have advocated and concrete efforts have been deployed towards the adoption of an international regulation to address the far-reaching consequences that cross-border reproduction can have on the human lives and rights of all those involved.^93^ The Court’s Advisory Opinion—as well as its contentious jurisprudence—fits therefore into this wider, supra-national endeavour to identify ‘common solutions to avoid limping legal parentage’.^94^ At the same time, however, it is important not to lose sight of the other side of the coin and, therefore, to ask ourselves how far and how fast it is advisable and desirable for the Court to go in such a controversial arena. In its first Advisory Opinion, it is by reading Article 8 as implying the possibility for recognition, but not of a specific type, that the Court manages to advance the interpretation of the Convention as a ‘living instrument’ whilst respecting (some) national variations.

## Footnotes

1 Cour de Cassation, Assemblée plénière, No 648 of 4 October 2019.

2 ECtHR [GC], *Advisory Opinion Concerning the Recognition in Domestic Law of a Legal Parent-Child Relationship Between a Child Born Through a Gestational Surrogacy Arrangement Abroad and the Intended Mother*, Request no P16-2018-001 by the French Court of Cassation, 10 April 2019.

3 Protocol No 16 to the Convention on the Protection of Human Rights and Fundamental Freedoms, 2 October 2013 (entry into force on 1 August 2018) <https://www.echr.coe.int/Documents/Protocol_16_ENG.pdf> accessed 19 October 2019.

4 J Jahn, ‘Normative Guidance from Strasbourg Through Advisory Opinions – Deprivation or Relocation of the Convention’s Core?’ (2014) 74 Zeitschrift für ausländisches öfentliches Recht und Völkerrecht 821, 823.

5 GP Smith II, ‘Judicial Decisionmaking in the Age of Biotechnology’ (1999) 13 Notre Dame Journal of Law, Ethics and Public Policy 93, 118.

6 C Byk, ‘Courts as Actors of Policy Making in Bioethics’ (2006) 17(1–2) Journal International de Bioétique 55.

7 Smith II (n 5) 94; A Margaria, *Nuove Forme di Filiazione e Genitorialità: Leggi e Giudici di Fronte alle Nuove Realtà* (Il Mulino 2018) 267–69.

8 JM Scherpe, *European Family Law – The Present and the Future of European Family Law* (Edward Elgar Publishing 2016) 18.

9 L Poli, ‘Il primo (timido) parere consultivo della Corte europea dei diritti umani: ancora tante questioni aperte sulla gestazione per altri’ (2019) 13(2) Diritti Umani e Diritto Internazionale 418, 420.

10
*Knecht v Romania*, No 10048/10, para 59, 2 October 2012.

11 For example, *Vo v France* [GC], No 53924/00, para 82, ECHR 2004-VIII; *Parrillo v Italy* [GC], No 46470/11, para 180, ECHR 2015.

12 J Gerards and E Brems, ‘Introduction’ in J Gerards and E Brems (eds), *Shaping Rights in the ECHR – The Role of the European Court of Human Rights in Determining the Scope of Human Rights* (CUP 2014) 1–3.

13 Protocol No 16 (n 3) art 1.

14 Brighton Declaration, ‘High Level Conference on the Future of the European Court of Human Rights’ (18–20 April 2012) <https://www.echr.coe.int/Documents/2012_Brighton_FinalDeclaration_ENG.pdf> accessed on 5 October 2019.

15 ibid, para 12(d); Protocol No 16 (n 3) Preamble.

16 K Dzehtsiarou and N O’Meara, ‘Advisory Opinion and the European Court of Human Rights: A Magic Bullet for Dialogue and Docket-Control?’ (2014) 34(3) Legal Studies 444, 451.

17
*Mennesson v France*, No 65192/11, ECHR 2014 (extracts).

18 RF Storrow, ‘The Phantom Children of the Republic: International Surrogacy and the New Illegitimacy’ (2012) 20(3) Journal of Gender, Social Policy and the Law 561, 565.

19 BC Van Beers, ‘Is Europe “Giving in to Baby Markets?” Reproductive Tourism and the Gradual Erosion of Existing Legal Limits to Reproductive Markets’ (2015) 23(1) Medical Law Review 103, 105.

20
*Mennesson* (n 17) paras 87–94.

21 ibid, para 96.

22 ibid, para 100. See also *Labassee v France*, No 65941/11, 26 June 2014; *Foulon and Bouvet v France*, Nos 9063/14 and 10410/14, 21 July 2016; *Laborie v France*, No 44024/13, 19 January 2017.

23 C Fenton-Glynn, ‘International Surrogacy Before the European Court of Human Rights’ (2017) 13(3) Journal of Private International Law 546, 555.

24 Cour de Cassation, Assemblée plénière, Nos 619 and 620 of 3 July 2015.

25 Cour de Cassation, 1ère Chambre Civile, Nos 824–827 of 5 July 2017.

26 ibid.

27 Advisory Opinion (n 2) para 9.

28 ibid, para 25; Explanatory Report to Protocol No 16 to the Convention for the Protection of Human Rights and Fundamental Freedoms, para 11.

29 ibid.

30 Advisory Opinion (n 2) para 25.

31 ibid.

32 ibid, para 26.

33 ibid, para 29.

34 ibid, para 28.

35 ibid, para 30.

36 ibid, para 37.

37 ibid, para 39 referring to *Mennesson*(n 17) para 99.

38 ibid, para 40.

39 ibid, para 41.

40 ibid, para 42.

41 L Lavrysen, ‘The Mountain Gave Birth to a Mouse: The First Advisory Opinion under Protocol No, 16’ (*Strasbourg Observers*, 14 April 2019) <https://strasbourgobservers.com/2019/04/24/the-mountain-gave-birth-to-a-mouse-the-first-advisory-opinion-under-protocol-no-16/> accessed on 20 October 2019.

42 R Spano, ‘Universality or Diversity of Human Rights? Strasbourg in the Age of Subsidiarity’ (2014) 14(3) Human Rights Law Review 487.

43 Advisory Opinion (n 2) para 43.

44 ibid.

45 ibid, para 45.

46 ibid, para 46.

47 ibid, para 47.

48 ibid, para 49.

49 ibid, para 51.

50 ibid.

51 ibid, para 52.

52 ibid, para 53.

53 ibid, para 55. See also C and E v France (Dec.), Nos 1462/18 and 17348/18, 12 December 2019.

54 Lavrysen (n 41).

55 ibid.

56 For example, AM Lecis Cocco Ortu, ‘L’Obbligo di Riconoscimento della Genitorialità Intenzionale tra Diritto Interno e CEDU: Riflessioni a Partire dal Primo Parere Consultivo della Corte EDU su GPA e Trascrizioni’ 2019 (1) Genius 68, 80.

57 Cour de Cassation (n 1).

58 ibid, para 19.

59 Y Ergas, ‘Thinking “Through” Human Rights: The Need for a Human Rights Perspective with Respect to the Regulation of Cross-border Reproductive Surrogacy’ in K Trimmings and P Beaumont (eds), *International Surrogacy Agreements: Legal Regulation at the International Level* (Hart 2013) 428.

60 ibid 431.

61
*Paradiso and Campanelli v Italy*, No 25358/12, 27 January 2015.

62
*Paradiso and Campanelli v Italy* [GC], No 25358/12, 24 January 2017.

63 Advisory Opinion (n 2) para 26.

64 ibid, para 36.

65 Lavrysen (n 41).

66 ibid.

67 Explanatory Report (n 28) para 11.

68 Brighton Declaration (n 14) para 35(c).

69 Jahn (n 4) 823.

70 J Christoffersen, ‘Individual and Constitutional Justice: Can the Power Balance of Adjudication be Reversed?’ in J Christoffersen and MR Madsen (eds), *The European Court of Human Rights between Law and Politics* (OUP 2011) 182.

71
*Konstantin Markin v Russia* [GC], No 30078/06, para 89, ECHR 2012 (extracts).

72 L Wildhaber, ‘A Constitutional Future for the European Court of Human Rights’ (2002) 23(5–7) Human Rights Law Journal 161.

73 ibid 163.

74 L Wildhaber, ‘The Role of the European Court of Human Rights: An Evaluation’ (2004) Mediterranean Journal of Human Rights 9, 28.

75 ECtHR Reflection Paper on the Proposal to Extend the Court’s Advisory Jurisdiction, para 5.

76 P Deumier and H Fulchiron, ‘Première demande d’avis à la CEDH: vers une jurisprudence ‘augmentée’’?’ (2019) (4) Recueil Dalloz 228, 231.

77 AS Brun-Wauthier and G Vial, ‘Gestation pour Autrui: Le Cercle Vertueux du Dialogue des Juges – A propos de l’avis consultatif de la CourEDH du 10 avril 2019’ (2019) 22 Revue des Droits et Libertés Fondamentaux <http://www.revuedlf.com/personnes-famille/gestation-pour-autrui-le-cercle-vertueux-du-dialogue-des-juges-a-propos-de-lavis-consultatif-de-la-couredh-du-10-avril-2019/> accessed 7 October 2019.

78 Jahn (n 4) 829.

79 This expression is taken from T Murphy, ‘Judging Bioethics and Human Rights’ in MK Land and JD Aronson (eds), *New Technologies for Human Rights Law and Practice* (CUP 2018) 87.

80 A Margaria, *The Construction of Fatherhood: The Jurisprudence of the European Court of Human Rights* (CUP 2019) ch 3.

81 ibid 69.

82 L Bracken, ‘Assessing the Best Interests of the Child in Cases of Cross-border Surrogacy: Inconsistency in the Strasbourg Approach?’ (2017) 39(3) Journal of Social Welfare and Family Law 368, 373.

83
*Mennesson* (n 17) para 100.

84
*Paradiso and Campanelli* (n 62) para 157.

85 M Ní Shúilleabháin, ‘Surrogacy, System Shopping, and Article 8 of the European Convention on Human Rights’ (2019) 33 International Journal of Law, Policy and the Family 104, 108.

86
*Paradiso and Campanelli* (n 62) para 195.

87 Advisory Opinion (n 2) para 36.

88 For example, *Re TT and YY* [2019] EWHC 1823 (Fam).

89 Ní Shúilleabháin (n 85) 108–09.

90 Explanatory Report (n 28) para 27.

91 K Lõhmus, *Caring Autonomy: European Human Rights Law and The Challenge of Individualism* (CUP 2015) 2–3.

92 W Müller-Freienfels, ‘The Unification of Family Law’ (1968) 16(1/2) American Journal of Comparative Law, 175, 175.

93 For example, K Trimmings and P Beaumont, ‘The European Court of Human Rights in *Paradiso and Campanelli v Italy* and the Way Forward for Regulating Cross-border Surrogacy’ (2017) University of Aberdeen Working Paper No 2017/3 <https://www.abdn.ac.uk/law/documents/CPIL%20Working%20Paper%20No%202017_3.pdf> accessed 19 October 2019; R Keating, ‘Left in Limbo: The Need to Regulate International Surrogacy Agreements’ (2014) 17 Trinity College Law Review 64, 91–92.

94 Hague Conference on Private International Law, *Report of the Experts’ Group on the Parentage/Surrogacy Project (Meeting of 28 January–1 February 2019)* (2019) pt 2 <https://assets.hcch.net/docs/55032fc1-bec1-476b-8933-865d6ce106c2.pdf> accessed 20 October 2019.

